# Effects of pelvic obliquity and limb position on radiographic leg length discrepancy measurement: a Sawbones model

**DOI:** 10.1186/s40634-022-00506-7

**Published:** 2022-07-26

**Authors:** Mohammed Nazmy Hamad, Isaac Livshetz, Anshum Sood, Michael Patetta, Mark H. Gonzalez, Farid A. Amirouche

**Affiliations:** 1 Department of Orthopedic Surgery, University of Illinois Chicago College of Medicine, 835 S. Wolcott Avenue, Chicago, IL 60612 USA; 2 Orthopedic Surgery, White Plains Hospital Physician Associates, White Plains, NY 10605 USA

**Keywords:** Leg length discrepancy, Lateral pelvic obliquity, Postural positional malalignment, Femoral abduction adduction, Radiographic measurement error, Total hip arthroplasty THA

## Abstract

**Purpose:**

Potential sources of inaccuracy in leg length discrepancy (LLD) measurements commonly arise due to postural malalignment during radiograph acquisition. Preoperative planning techniques for total hip arthroplasty (THA) are particularly susceptible to this inaccuracy, as they often rely solely on radiographic assessments. Owing to the extensive variety of pathologies that are associated with LLD, an understanding of the influence of malpositioning on LLD measurement is crucial. In the present study, we sought to characterize the effects of varying degrees of lateral pelvic obliquity (PO) and mediolateral limb movement in the coronal plane on LLD measurement error (ME).

**Methods:**

A 3-D sawbones model of the pelvis with bilateral femurs of equal-length was assembled. Anteroposterior pelvic radiographs were captured at various levels of PO: 0°, 5°, 10°, and 15°. At each level of PO, femurs were individually rotated medio-laterally to produce 0°, 5°, 10°, and 15° of abduction/adduction. LLD was measured radiographically at each position combination. For all cases of PO, the right-side of the pelvis was designated as the higher-side, and the left as the lower-side.

**Results:**

At 0° PO, 71% of tested variations in femoral abduction/adduction resulted in LLD ME < 0.5-cm, while 29% were ≥ 0.5-cm, but < 1-cm. ME increased progressively as one limb was further abducted while the contralateral limb was simultaneously further adducted. The highest ME occurred with one femur abducted 15° and the other adducted 15°. Similar magnitudes of ME were seen in 98% of tested femoral positions at 5° of PO. The greatest ME (~ 1 cm) occurred at the extremes of right-femur abduction and left-femur adduction. At 10° of PO, a higher prevalence of cases exhibited LLD ME > 0.5-cm (39%) and ≥ 1-cm (8%). The greatest errors occurred at femoral positions similar to those seen at 5° of PO. At 15° of PO, half of tested variations in femoral position resulted in LLD ME > 1-cm, while 22% of cases produced errors > 1.5-cm. These clinically significant errors occurred at all tested variations of right-femur abduction, with the left-femur in either neutral position, abduction, or adduction.

**Conclusion:**

This study aids surgeons in understanding the magnitude of radiographic LLD ME produced by varying degrees of PO and femoral abduction/adduction. At a PO of ≤5°, variations in femoral abduction/adduction of up to 15° produce errors of marginal clinical significance. At PO of 10° or 15°, even small changes in mediolateral limb position led to clinically significant ME (> 1-cm). This study also highlights the importance of proper patient positioning during radiograph acquisition, demonstrating the need for surgeons to assess the quality of their radiographs before performing preoperative templating for THA, and accounting for PO (> 5°) when considering the validity of LLD measurements.

## Introduction

Total hip arthroplasty (THA) is recognized as a reliable intervention usually performed to relieve pain, optimize hip mobility and stability, and improve patient function [1, 2]. Maintaining or restoring equal limb length during this procedure is key for achieving optimal hip biomechanics and patient satisfaction [3, 4].

Although the boundary between acceptable and unacceptable levels of leg length discrepancy (LLD) after THA remains undefined, it’s generally accepted that LLD magnitudes greater than 15–20 mm are perceivable by patients, lead to poorer functional outcomes and patient dissatisfaction [5, 6], and are associated with a higher incidence of adverse effects [7, 8]. These include compensatory gait abnormalities [9, 10], sciatic, femoral, or peroneal nerve palsies, chronic lower back pain, and degenerative arthritides of the lower extremities and lumbar spine [11, 12]. Some adverse manifestations, including neuropathic pain and LLD-induced motor deficits have been shown to improve following equalization of limb length [13, 14]. To achieve the most favorable outcomes and prevent potential complications, it is generally suggested that surgeons aim for a LLD of less than 10 mm during THA [15, 16]. All these factors highlight the importance of obtaining proper limb length measurements pre- and intra-operatively.

To minimize the risk of postoperative LLD, most surgeons consider preoperative templating to be crucial for planning a successful THA [17]. Templating is typically performed on anteroposterior (AP) pelvic radiographs and allows surgeons to reliably predict the size of the prosthesis required and the appropriate amount of offset, as well as, to anticipate for any necessary limb length restoration [17–19]. While several preoperative templating methods have been described in the orthopedic literature, the ready-availability and low cost of AP pelvic radiography makes it the most widely used tool and the method-of-choice for THA [20]. Although clinicians are not united in regard to the most accurate LLD measurement technique as applied on pelvic radiographs [21, 22], LLD is most often determined as the distance between a line passing through the teardrop points medial to the acetabula or the ischial tuberosities to the tip of the lesser trochanter [21, 23].

Based on etiology, LLD can be conceptualized as falling into one of two main categories: 1) structural or ‘true’ LLD and 2) functional or ‘apparent’ LLD [24, 25]. True LLD is attributable to actual shortening of bony structures, usually as a result of defective pelvic or femoral anatomy. In contrast, apparent LLD implies no actual bony defect is present, but instead is the result of altered biomechanics of the lower limbs. Clinically, apparent LLD is associated with postural asymmetry and is often caused by soft-tissue contractures or PO [26]. Moreover, apparent LLD is considered to be the measure of the limb length inequality that is actually perceivable by the patient. In some instances, both structural and functional LLDs can be present simultaneously. In such cases, the discrepancies may amplify or balance one another, depending on the sides involved.

In the present study, we explore the relationship between PO, femoral positioning, and LLD measurement using a three-dimensional model. We characterize and quantify the effects of increasing degrees of PO and various amounts of medio-lateral femoral rotation on resulting LLD measurement error (ME). In the context of our study design, ME reflects the difference between known ‘true’ LLD and ‘apparent’ LLD, as determined by radiographic measurement. We explore the functional LLD introduced by postural malalignment and determine the postural parameters required to produce clinically significant errors in LLD measurement.

## Methods

### Hip model

**Fig. 1:**
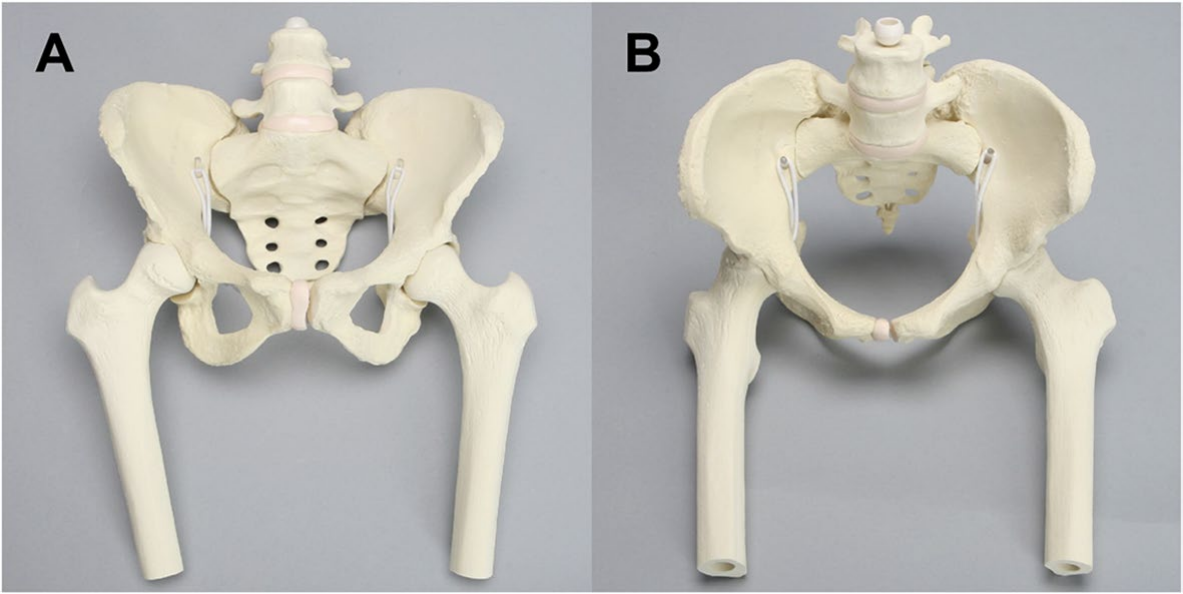
(**A**) Anterior and (**B**) superior view of the assembled Sawbones model, consisting of a pelvis and bilateral articulating acetabular joints. The composite model material is designed to mimic human cadaveric bone. The material’s radiopaque cortex allows capture of high-resolution radiographs. Femurs are equal in length bilaterally

A large-sized Sawbones model (Sawbones USA: Vashon Island, WA, United States) of the pelvis, with bilateral articulating femurs of equal length, was assembled to simulate normal hip structure (Fig. 1). Sawbones composite bone modeling material was chosen for its topographic similarity to human cadaver bone and its radiopaque cortical shell, allowing capture of high-resolution radiographic images.

**Fig. 2:**
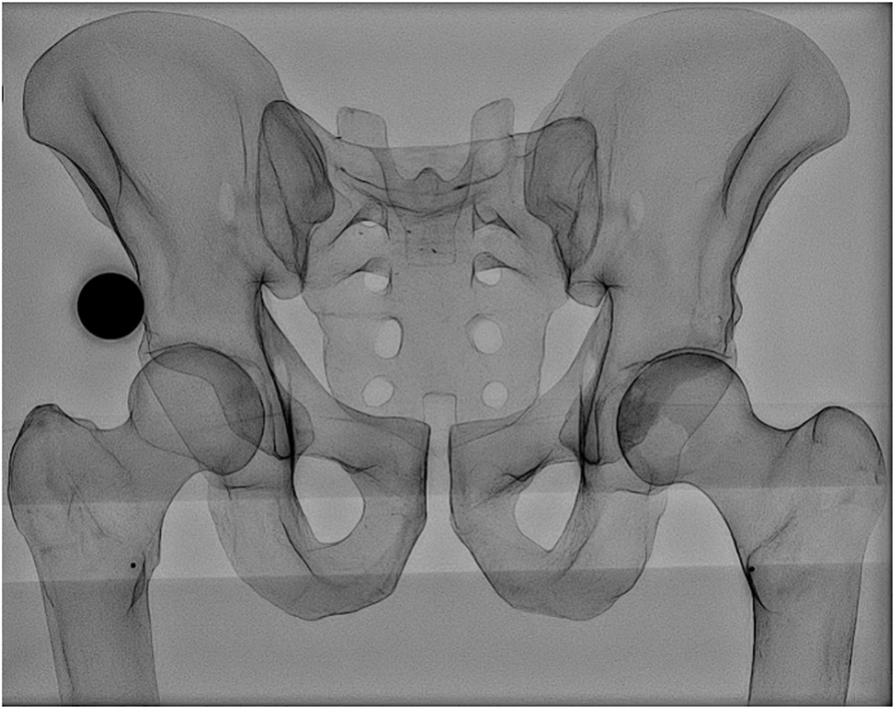
Anteroposterior (AP) radiograph of the Sawbones model, demonstrating its radiopaque cortical shell. For calibration and reference, a 25-mm steel ball is placed at the level of the acetabulofemoral joint and a 2-mm metallic marker at the lesser trochanter

**Fig. 3:**
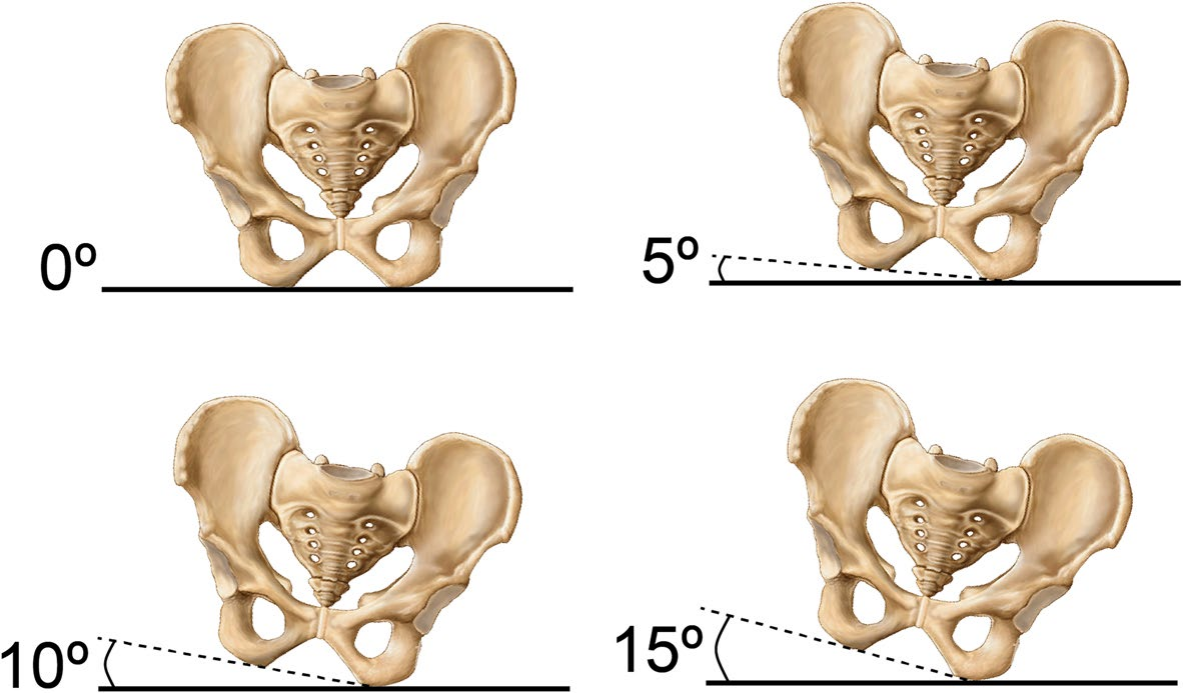
Shown are 2-dimensional pictorial representations of varying degrees of PO portrayed by views of the pelvis in the coronal plane. Depiction in this way simulates the AP radiographic view of the pelvis commonly used to measure LLD in the clinical setting. Each depiction demonstrates one of the four levels of PO assessed with our Sawbones model. To establish neutrality (PO of zero), a level horizontal axis bisecting the inferior aspects of the ischial tuberosities was established. All other levels of obliquity (5°, 10°, and 15°) were produced with reference to this horizontal axis. Note that for the purposes of our assessment, the right side of the pelvis is considered the higher side, while the left is considered the lower side. At each of the four pelvic obliquities shown, four levels of femoral abduction (0°, 5°, 10°, 15°) and adduction (0°, 5°, 10°, 15°) were evaluated

For calibration and reference purposes, a 25-mm steel ball was affixed to the model at the level of the hip joint and a 2-mm metallic marker was affixed at the level of the lesser trochanters, bilaterally (Fig. 2). Rapid sequential fluoroscopy images (R90 Fluoroscopy System, Philips, Amsterdam, Netherlands) were captured at various levels of PO and various degrees of femoral abduction and adduction with the model in supine position and femurs internally rotated 15°. The following levels of PO were assessed: 0° (neutral, level pelvis), 5°, 10°, and 15° (Fig. 3). At each level of obliquity, the left and right femurs were individually rotated medio-laterally in the coronal plane to produce 0°, 5°, 10°, and 15° of abduction and adduction. All combinations of left/right femoral abduction/adduction were modeled and captured radiographically for each level of PO, except those that occurred at the unlikely extremes of motion. These unlikely extremes occurred exclusively at pelvic obliquities of 10° and 15°. All in all, 49 radiographs each were obtained for pelvic obliquities of 0° and 5°, while 36 were obtained for obliquities of 10° and 15°. A total of 170 radiographs were captured, each representing a different combination of pelvic and femoral positioning.

Positioning of the model at each PO was based on a horizontal reference axis established with the pelvis in neutral position (Fig. 3), bisecting the inferior aspects of the ischial tuberosities. Medio-lateral rotation of each of the model femurs was based on a horizontal reference axis established by alignment of the centers of the femoral heads with the tips of the greater trochanter. This was considered the neutral femoral position, without any abduction or adduction. All levels of PO and femoral rotation were verified radiographically using image markup software (dicomPACS, OR Technology, Oehm und Rehbein, Germany). This served to validate that the Sawbones model was properly oriented for each of the evaluated postures.

### LLD Measurement & Error Reporting

Measurement of LLD was performed digitally using medical imaging annotation software (dicomPACS, OR Technology, Oehm und Rehbein, Germany). The bony landmarks used for measurement were based on Woolson et al’s. measurement technique [19]. By this method, a horizontal line is drawn bisecting the lower-edge of the acetabular teardrops, bilaterally (inter-teardrop line; ITL). Then, for each limb, the perpendicular distance between the ITL and the medial tip of the lesser trochanters (LT) is determined. The difference in measurement between the two limbs represents the LLD. LLD was measured using this method on all 170 radiographs obtained.

The Sawbones model we constructed was designed to have an inherent ‘true’ LLD of zero, given the bilateral equivalence of limb length. Therefore, the radiographically measured LLD values reflect the ‘apparent’ LLD introduced by the respective pelvic/femoral position being evaluated. The difference between this ‘apparent’ LLD and the ‘true’ LLD reflects the measurement error (ME). Since the ‘true’ LLD is simply zero, then the ‘apparent’ LLD is itself equal to the ME.

### Statistical analysis

Data analysis was performed using SPSS (SPSS Statistics for macOS, Version 24.0., IBM Corp.: Armonk, NY, United States) and Excel (Microsoft Excel for macOS, Version 16.47., Microsoft Corp.: Redmond, WA, United States). Measured outcomes were found to satisfy the conditions of normality, equal variance, and independence. Therefore, statistical analysis of study data was achieved using parametric statistical methods: independent sample t-testing and one-way analysis of variance (ANOVA). A *p*-value less than 0.05 was considered statistically significant.

## Results

A total of 170 radiographs were captured including: 49 at neutral pelvic position, 49 at a PO of 5°, 36 at an obliquity of 10°, and 36 at an obliquity of 15°.

**Fig. 4:**
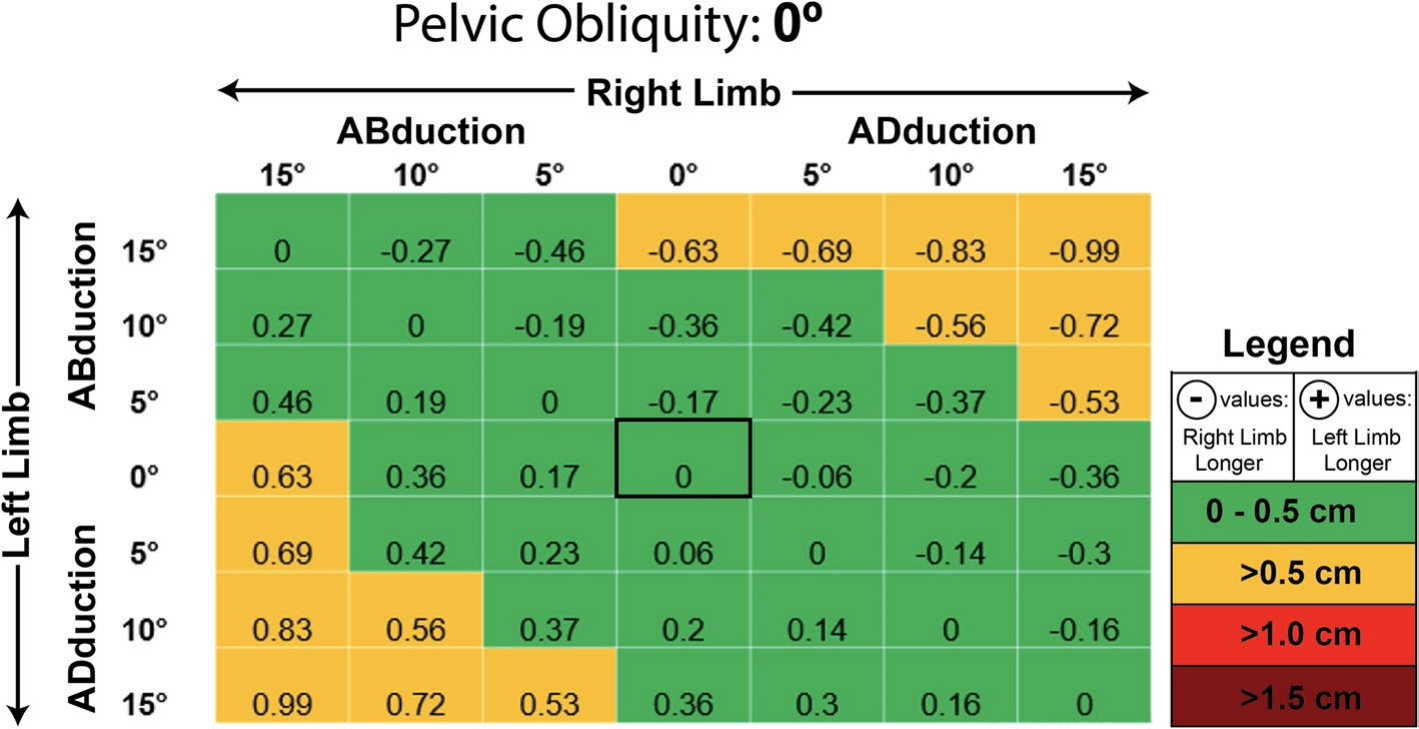
Measurement error in centimeters seen at varying limb positions when the pelvic is set to 0 degrees of lateral obliquity (level pelvis in neutral position)

**Fig. 5:**
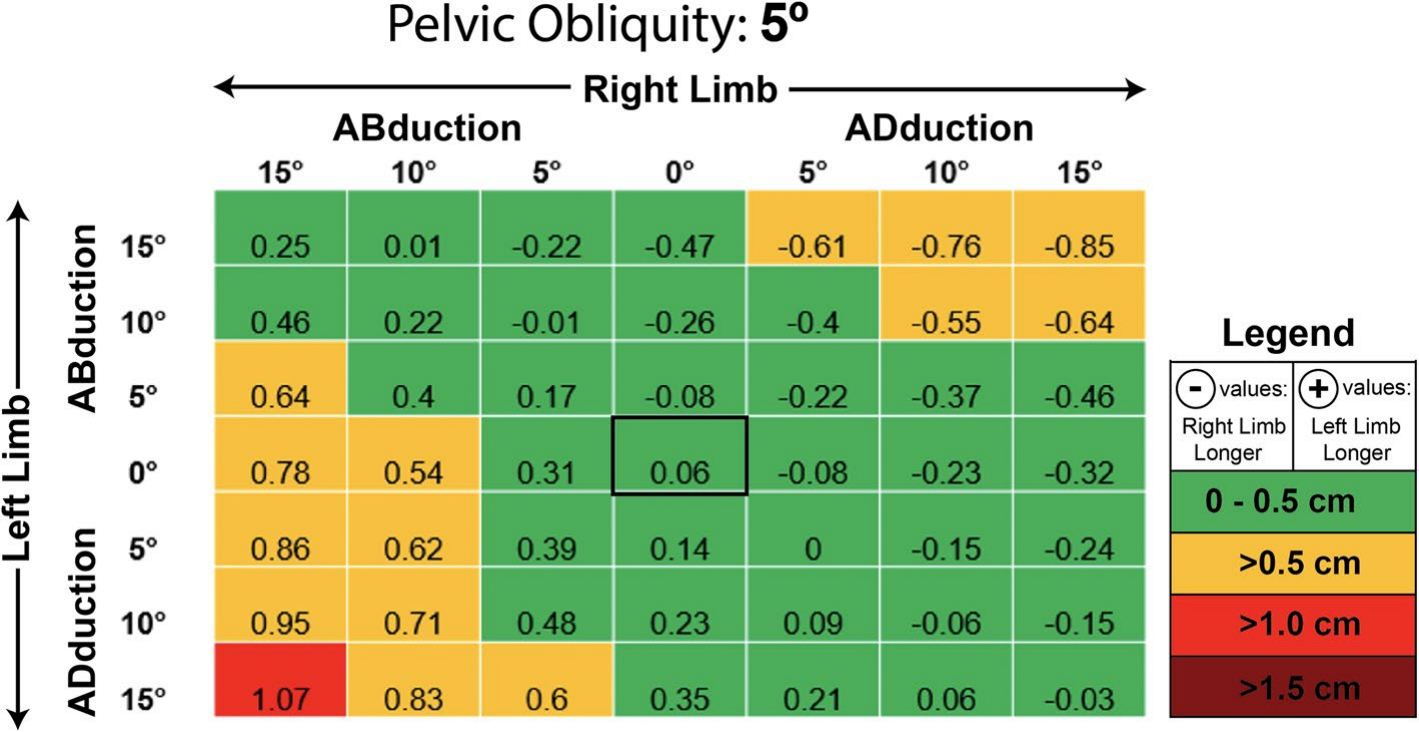
Measurement error in centimeters seen at varying limb positions when the pelvic is set to 5 degrees of lateral obliquity

At neutral pelvic position (Fig. 4), 35 of 49 tested variations in femoral abduction/adduction (~ 71%) resulted in LLD ME < 0.5 cm. The remaining 14 of 49 postural variations (~ 29%) were greater than 0.5 cm, however, none were ≥ 1 cm at a PO of 0°. Similar results were seen at a PO of 5° (Fig. 5), which demonstrated 34 of 49 cases (~ 69%) to have MEs < 0.5 and 14 of 49 (~ 29%) to have a MEs > 0.5 cm but < 1 cm. A single case (~ 2%) in which the limb on the lower side of the pelvis (left) was adducted 15°, and the contralateral limb on the higher side of the pelvis (right) was abducted 15° was found to produce a ME of 1.07 cm. Statistical analysis demonstrated that the LLD MEs found at PO = 5° (mean ME: 0.087 ± 0.467 cm) were not significantly different than the MEs seen at neutral pelvic position (obliquity = 0°; mean ME: 0.000 ± 0.448 cm), t (96) = − 0.943, *p* = 0.348. Overall, the MEs seen at a PO of 5° were approximately 98% similar to the MEs seen with a neutral pelvis.

**Fig. 6:**
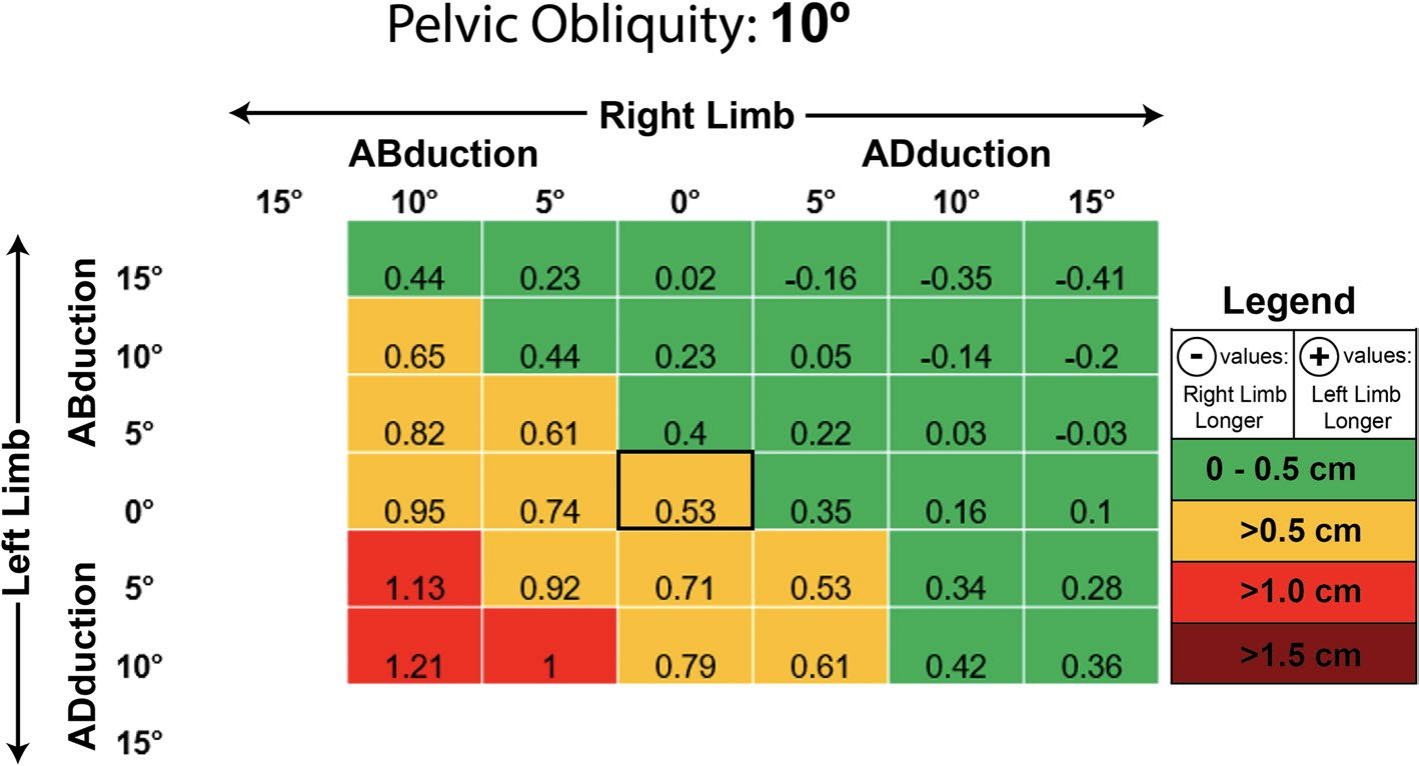
Measurement error in centimeters seen at varying limb positions when the pelvic is set to 10 degrees of lateral obliquity

At a PO of 10° (Fig. 6), a higher portion of the tested postural variations exhibited MEs > 0.5 cm (14 of 36 cases; ~ 39%) and > 1 cm (3 of 36 cases; ~ 8%). However, the majority of postural variations continued to produce MEs < 0.5 cm (22 of 36 cases; ~ 61%). The highest MEs occurred in cases in which the femur on the lower side of the pelvis (left) was adducted and the contralateral limb on the higher side of the pelvis was abducted. The increased MEs seen with the pelvis and femurs oriented in this way are similar to those seen at a PO of 5°. Statistical analysis demonstrated that the LLD MEs at PO = 10° (mean ME: 0.388 ± 0.410 cm) were significantly higher than the MEs seen at PO = 0°, t (83) = − 4.094, *p* = 0.000. Moreover, the LLD MEs occurring at PO = 10° were statistically significantly higher than those seen at PO = 5°, t (83) = − 3.093, *p* = 0.003.

**Fig. 7:**
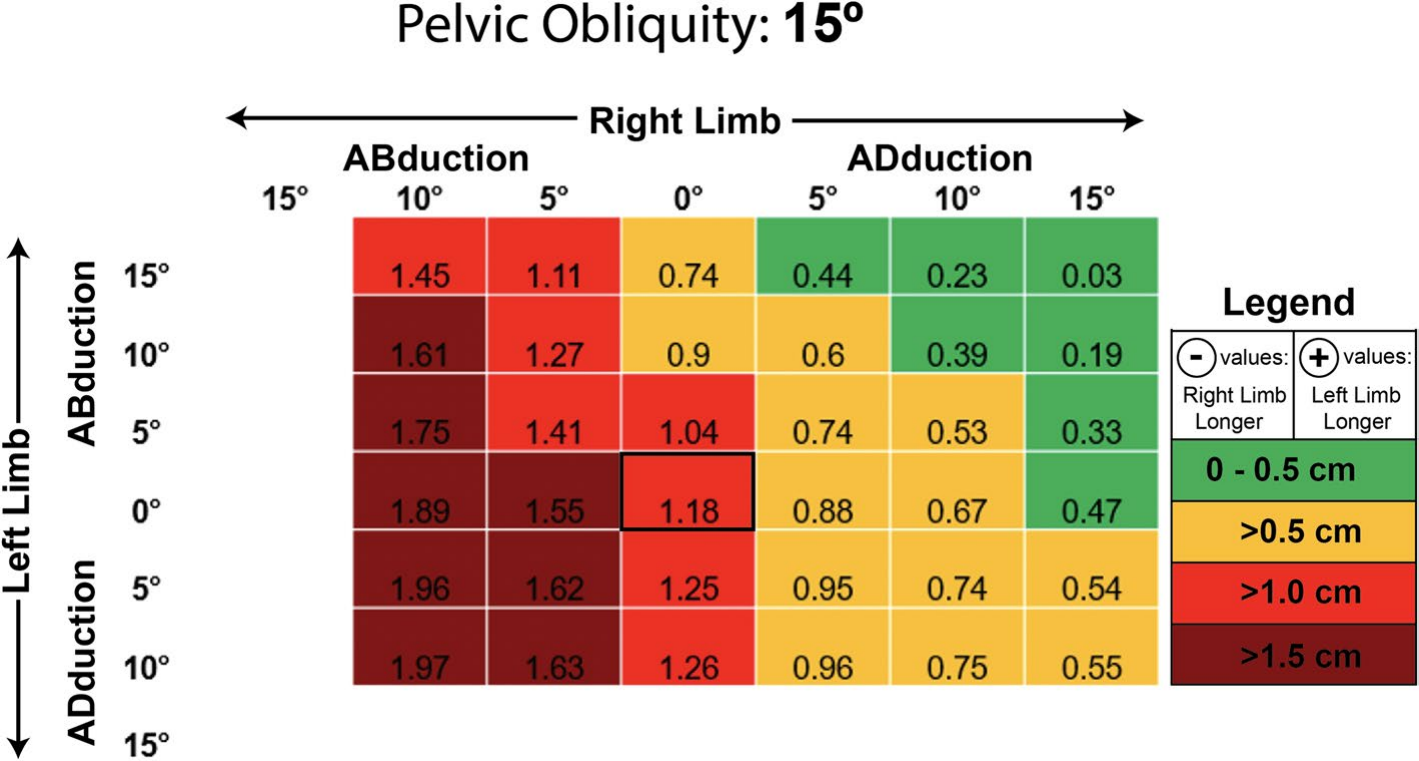
Measurement error in centimeters seen at varying limb positions when the pelvic is set to 15 degrees of lateral obliquity

At a PO of 15° (Fig. 7), 16 of 36 tested postural variations (~ 44%) resulted in a ME > 1 cm. Moreover, 8 of 36 cases (~ 22%) had a ME > 1.5 cm. Only 7 of 36 cases (~ 19%) had a ME < 0.5. As seen with prior pelvic obliquities, the highest MEs occurred when the limb of the higher-side of the pelvis (right) was abducted and the limb of the lower-side was adducted (left). Statistical analysis demonstrated that the LLD MEs at PO = 15° (mean ME: 0.988 ± 0.537 cm) were significantly higher than the MEs seen at PO = 0°, t (83) = − 9.240, *p* = 0.000. LLD MEs at PO = 15° were also statistically significantly higher than those seen at PO = 5°, t (83) = − 8.251, *p* = 0.000, and those seen at PO = 10°, t (70) = − 5.330, *p* = 0.000.

## Discussion

In the present study, we determined the extent of radiographic LLD ME introduced as a result of pelvic and femoral malposition. Clinically, the assessed variations in posture are usually a consequence of either intrinsic bony or soft tissue malformations, improper patient positioning during radiography, or by the simultaneous occurrence of both circumstances [27]. We demonstrate that, depending on the degree of lateral PO and femoral abduction/adduction present, ME may be significant enough to lead to the exhibition of clinically relevant apparent discrepancies, even when in actuality, none truly exists. By constructing a 3-dimensional artificial bone model with an inherent true LLD of zero, we were able to simulate postural variance and ascertain the resulting functional discrepancies that manifested on AP pelvic radiographs. By using this method, we’ve been able to quantitatively correlate degrees of pelvic and femoral postural variation with LLD ME, expressed as the difference between known true LLD and radiographically measured apparent LLD.

Four levels of coronal PO were assessed in our study: 0°, 5°, 10°, and 15°. These were chosen based on previously published works demonstrating the range of obliquities most commonly seen clinically in THA [28, 29]. Categorically, PO can be classified as suprapelvic, intrapelvic, and infrapelvic, depending on its origin. Suprapelvic obliquity results secondary to spinal pathology, such as scoliosis or degenerative disease of the lumbosacral spine, while intrapelvic obliquity results secondary to architectural bony defects inherent in the hemipelvis [27]. The latter often goes on to lead to ischial and ilium hypoplasia. Infrapelvic obliquity, the most common type implicated in THA cases, and the focus of this study, is considered to result secondary to abduction or adduction hip contractures or limb length inequalities [30]. It has been shown that most patients undergoing THA with radiographic evidence of infrapelvic obliquity do so with a severity ranging from 0° to 15°, with the majority of patients having an obliquity ≤10° [30–32].

As indicated by our results, assessments performed in which the pelvis was kept in neutral position (lateral obliquity of 0°) with the femurs simultaneously and individually abducted and adducted from 0° to 15° consistently demonstrated LLD MEs of marginal clinical significance (≤10 mm). Similar assessments, but with the pelvis laterally rotated to an obliquity of 5°, demonstrated comparable magnitudes of ME, with 98% of tested variations in femoral abduction and adduction resulting in apparent LLDs of marginal clinical significance. Predictably, these results were not maintained at increasing degrees of PO. At 10° and 15° of obliquity, even small changes in femoral position led to significant magnitudes of ME (> 10 mm), with the greatest errors occurring at the latter. While 8% of tested variations in femoral position resulted in clinically significant ME at 10° of PO, 44% of cases reached this threshold at 15° of obliquity. Interestingly, across all levels of PO, the greatest magnitudes of ME occurred at positions in which the femur of the high-side of the pelvis was abducted and the femur of the low-side of the pelvis was adducted. Assessment of the biomechanics associated with this position allows us to shed light on why this may have been the case. The high-side of the pelvis can be considered as one that is affected by an adduction contracture. Alternatively, the low-side of the pelvis can be considered as having an abduction contracture. In either case, this results in the high-side of the pelvis appearing functionally shorter in leg length, while the low-side of the pelvis appears functionally longer [33]. The effects of femoral position (adduction or abduction) may either magnify or offset the hip’s effects on leg length. As shown by Sarin et al., 2005 and again more recently by Kawamura et al., 2021, femoral adduction can lead to functional limb lengthening, while femoral abduction can lead to functional limb shortening [34, 35]. In our case, femoral abduction compounds the shortening effects of the pelvic adduction contracture on the high-side of the pelvis. This maximizes the magnitude of functional limb shortening which is achievable by manipulation of pelvic and femoral structures in the constraints of this study’s parameters. On the contralateral side (low-side) of the pelvis, femoral adduction magnifies the lengthening effects of the pelvic abduction contracture, thus producing a maximally lengthened apparent limb.

The results of this study highlight the importance of achieving proper pelvic and femoral positioning during capture of radiographs. Moreover, our results stress that when PO is present on radiographs, it’s paramount that orthopedic surgeons take this into account when measuring LLD, especially when obliquity is greater than 5°. This is important to consider pre-, intra-, and post-operatively, particularly in the context of planning, performing, and following-up THA procedures.

Radiographs are the most commonly used modality in the evaluation of LLD before and after THA, with measurements usually achieved on AP views of the pelvis and proximal femurs [23]. Although several other methods for assessing LLD have been described, their use is often limited by inherent disadvantages in accuracy, reliability, susceptibility to magnification error, radiation exposure, cost, need for special equipment, or overall inconvenience [36]. For instance, clinical methods, such as those involving use of a tape measure and standing blocks, have consistently been found to be less accurate compared to conventional imaging modalities. Despite this, clinical methods have been noted to be useful screening tools [37–40]. In addition to plain radiography, other proposed imaging modalities for achieving LLD measurement include computed radiography, slit scanograms, microdose digital radiography, CT scanograms, and MRI scans [36]. Each has their own advantages and disadvantages, a full description of which is outside the scope of this article.

**Fig. 8:**
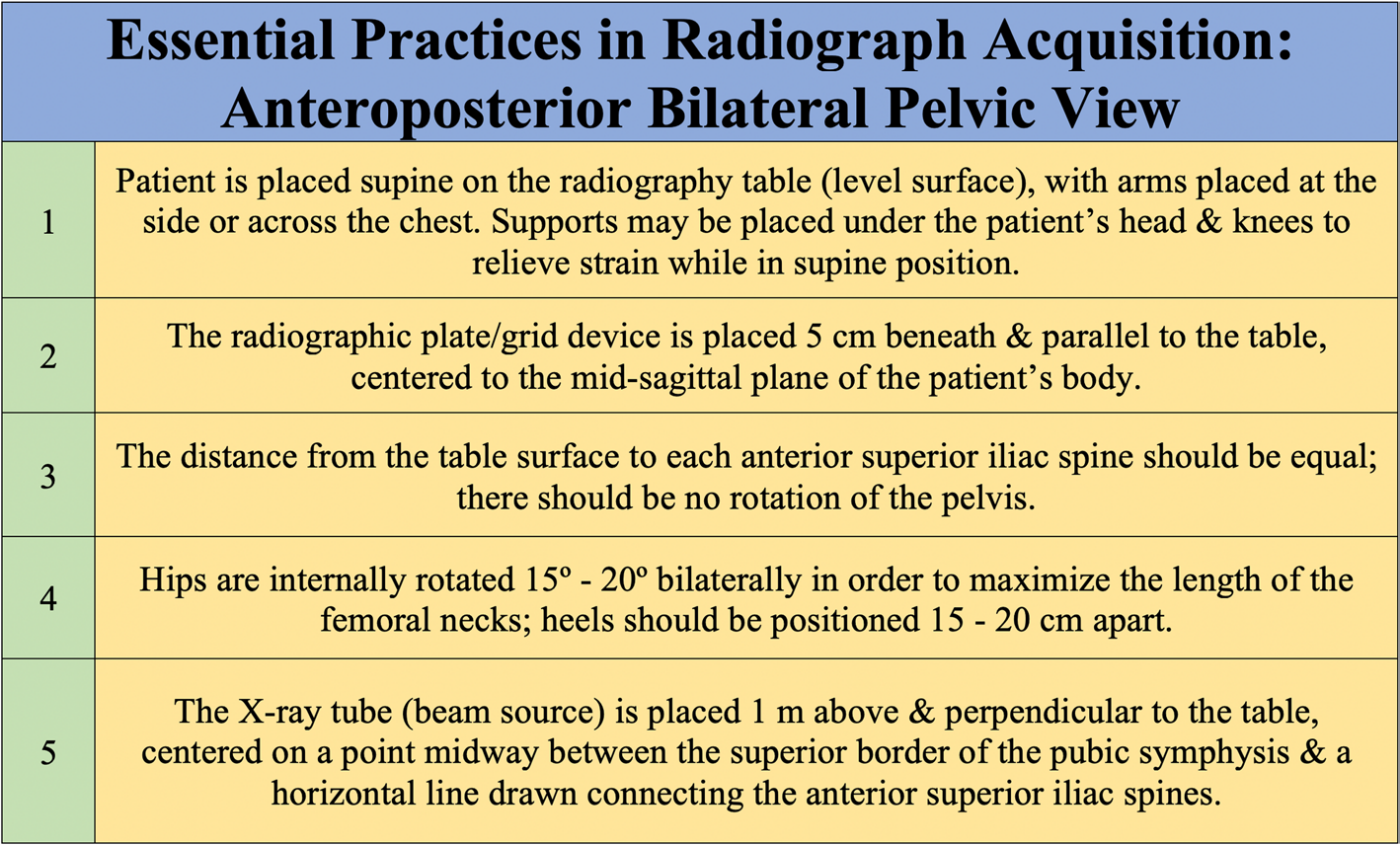
Supine AP pelvic radiographs are routinely used for planning hip arthroplasties The most crucial aspects of proper radiograph acquisition for this projection are described, with special focus on patient and equipment positioning. We focus on supine rather than standing AP pelvic radiographs given the practical considerations relevant to performing THA -- supine radiographs can be compared directly to pelvic radiographs captured intra-operatively or at early follow-up when the patient may have restricted weight bearing.

**Fig. 9:**
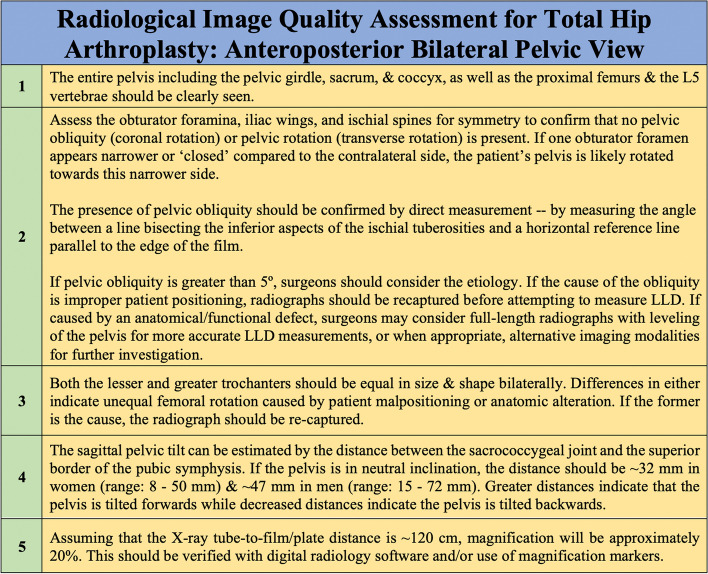
The significance of properly reviewing radiographs and assessing their quality before carrying our measurements such as LLD cannot be understated. A method for assessing AP pelvic radiographs, including practical considerations for recognizing PO and other postural malalignments is described. The five quintessential radiographic properties outlined in this figure should all be satisfied before deciding to accept a pelvic radiograph as sufficient for producing accurate LLD measurements or for use in preoperative templating for THA

**Fig. 10:**
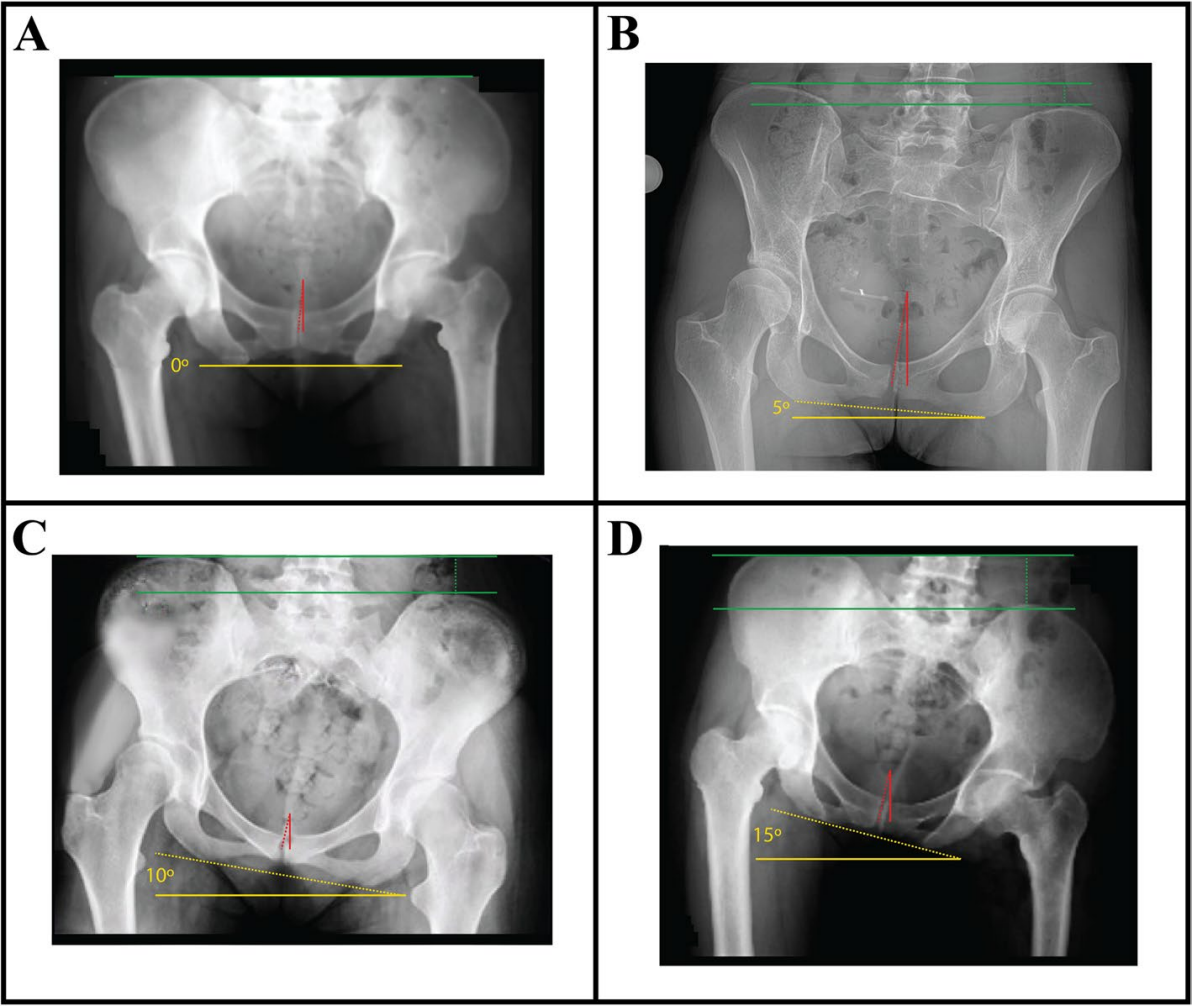
**A** An AP pelvic radiograph of a patient with a level pelvis is shown. Notice the relative bilateral symmetry in the size and shape of the obturator foramina, the iliac wings, the ischial spines, and the ischial tuberosities. This symmetry, viewed in context of the relative vertical alignment of coccyx and the midline of the pubic symphysis gives us an overall impression of a lack of PO. **B** A PO of 5° is seen in this radiograph. Notice the bilaterally asymmetric heights of the iliac crests and the relatively increased vertical malignment of the tip of the coccyx and the midline of the pubis compared to the neutral pelvis. **C** At a PO of 10°, the asymmetry between the bilateral obturator foramina and the heights of the iliac crest become much more profound. **D** Demonstrating even greater postural asymmetry, this radiograph shows a PO of 15°

To accurately and reliably determine LLD via radiographic measurement on AP views of the pelvis, radiographs must always be obtained using validated and standardized imaging protocols, whenever possible [41]. Error-free LLD measurement is highly dependent on image quality, which itself is highly dependent on both technique and patient positioning. Variability in the latter can substantially impact a surgeon’s ability to detect, diagnose, and quantify structural abnormalities. AP pelvic radiographs can be obtained with the patient in standing, weight-bearing position or in supine position. Cleveland et al. previously compared LLD measurements achieved using both standing and supine pelvic radiographs and, using 10 mm as the threshold for meaningful difference, reported no difference between the two tests [42]. Despite this, surgical planning for THA has traditionally preferred supine AP radiographic images of the pelvis. Standing images, when obtained, are typically used as an adjunct and not a replacement for supine imaging [43]. This is largely attributable to the significant differences in pelvic tilt and acetabular anteversion and inclination seen between supine and standing positions [43]. If tilt or rotation of the pelvis is evident with the patient lying supine, suspicion and diagnosis of hip contracture or lumbosacral pathology is more readily established [27]. The ideal AP radiographic view captures the top of the iliac crests and extends distally, just beyond the lesser trochanters. With the patient lying in the supine position on a level surface, most standardized protocols direct that the patient’s hips be internally rotated 15° to 20°. This ensures a view in which the pelvis and femoral neck are forward-facing. This is particularly significant for the latter, as the femoral neck has a normal anteversion of 15° to 20°. If radiographs are obtained with the hips externally rotated, a gross underestimation of femoral offset may result [27]. Typical technique also dictates that the X-ray tube be placed 1 m above and oriented perpendicularly to the table, and the radiographic plate/film be placed 5 cm beneath the table. This allows for a magnification of ~ 20% (± 6%) [44]. Magnification is directly proportional to the distance between the pelvis and the radiographic plate/film. For adequate preoperative planning, surgeons need to know the magnification of the hip radiographs, so as to account for it when templating and establishing their measurements. Increased magnification should be expected and accounted for in obese patients, and conversely reduced magnification for thin patients [27]. For patients requiring absolute precision, magnification markers of known diameter can be positioned along the patient’s coronal plane at the level of the greater trochanter [45]. The X-ray beam itself is commonly centered on a point midway between the superior border of the patient’s pubic symphysis and a line drawn connecting the anterior superior iliac spines [43, 46]. Lowering the X-ray beam to be aligned centered near the pubis ensures that the whole proximal third of the femurs are visible and are more or less located in the same horizontal plane as the X-ray source. This prevents excessive distortion in the resulting image. Considering the many factors involved in radiograph acquisition, it’s not surprising that obtaining an ideal image can be challenging in clinical practice, particularly in patients who present with inherent bony or soft tissue abnormalities causing intrinsic postural variation that deviates significantly from normal anatomy. A summary of the proper techniques for capturing supine AP pelvic radiographs is outlined in Fig. 8. The described procedure focuses on proper patient positioning and the relative arrangement of radiological equipment required to successfully capture a pelvic view at the commonly used magnification of ~ 20%. Figure 9 describes a method by which surgeons are able to review and assess the quality of their radiographs before accepting them as adequate for planning a THA and using them for LLD measurements. At its core, this method entails assessment of femoral rotation, sagittal pelvic inclination, coronal and transverse pelvic rotation, and pelvic/femoral symmetry. Many of the radiographical features described in the method can be seen in Fig. 10, which demonstrates various levels of PO ranging from 0° to 15° on AP pelvic radiographs.

The widespread use of pelvic radiography for preoperative templating and determining LLD emphasizes the significance of the present study. Given the commonplace occurrence by which pelvic and femoral malpositioning is present on captured radiographs, it’s important that surgeons understand the potential measurement errors introduced by varying degrees of lateral obliquity and mediolateral femoral rotation. Only by recognizing the magnitudes of error involved can surgeons adequately determine whether the degree of postural variation is within acceptable limits, or whether radiographs should be recaptured, alternative views be obtained, or whether alternative imaging modalities are warranted.

While the results of this study have promising implications, it was subject to several limitations. Firstly, given that a single model was used to simulate pelvic and femoral position, the generalizability of the magnitudes of ME is limited. Despite this, the relative measurements we obtain with each of the tested postural variations remains valid. Future studies could potentially explore LLD ME introduced by more complex models which better replicate the functional deformities that are commonly seen in clinical practice. The importance of this is highlighted by the fact that ME is proportional to patient size and varies depending on the relative distance between the lesser trochanters and the centers of the femoral heads. An investigation using more diverse models would serve to strengthen the applicability of our results. Secondly, we chose to construct a model with a true LLD of zero in order to facilitate determination of ME and introduced apparent LLD. A more comprehensive characterization of the magnitudes of error detected at each posture would be possible using models with preexisting true LLD. By overcoming this limitation, studies would be able to expound on any potential compounding effect that, taken together, may worsen or lessen the overall ME. Finally, this study was limited in that it examined only two parameters of postural variation, both occurring in the coronal plane: lateral PO and mediolateral femoral abduction and adduction, and both measured on a single radiographic view: supine bilateral AP pelvis. The effects of posteroanterior pelvic tilt and pelvic rotation in the transverse plane were not assessed, although they are commonly implicated in radiographic malpositioning. Moreover, discrepancy arising from the variations in the femoral shaft, knee, tibia, or ankle were also not accounted for.

We’ve seen that the LLD depicted on any given AP pelvic radiograph is dependent on the pelvic orientation of the subject during X-ray acquisition. We’ve also demonstrated that non-neutral variations in PO can directly alter LLD measurements to a clinically relevant degree, and that the magnitude of the introduced error is directly related to the degree of PO. Based on these findings, we may now also consider --- what other measurable radiographic parameters are quantitatively or qualitatively altered to a clinically relevant degree when subject to variations in PO? Can these changes influence the diagnosis of acetabular pathologies or adversely affect surgical planning? Unfortunately, a non-ambiguous characterization of this highly-relevant topic remains elusive, and the answers to these questions vary significantly depending on the parameter and pathology under consideration. Underlying this are conflicting results between studies, small study sizes, and a lack of standardized widely-accepted investigation techniques [47–51]. For instance, in an experimental setup involving 20 cadaveric pelvises, Tannast et al. ventured to explore the effects of pelvic tilt (ranging from − 24° to 24°) and PO (ranging from − 12° to 12°) on 11 radiographic parameters [50]. These investigators found no quantitatively relevant change in the following parameters as they varied PO: lateral center-edge angle (LCEA), acetabular index (AI), extrusion index (EI), ACM angle, Sharp angle, and cranio-caudal coverage. In contrast, they found that anteroposterior acetabular coverage, crossover and posterior wall signs, and retroversion index exhibited clinically relevant changes. In a similar cadaveric study, Monazzam et al. found that PO significantly affected LCEA, AI, and Sharp angle measurements -- contradicting Tannast and colleagues results [22]. Currently, although there is an abundance of articles within the orthopedic literature which explore and characterize the effects of pelvic tilt on radiographic parameters, significantly fewer articles explore the effects of PO [52–55].

Future research focusing not only on more complex and diverse modeling, but also on validating our results in vivo with patients of varying sizes and functional deformities would be well suited for expanding our understanding of the effects of postural variation on radiographic measurement. Assessments of other parameters in addition to LLD by the same manner would be beneficial. By comprehensively characterizing and quantifying the effects of postural variation, algorithm-based software applications which calculate corresponding ME become within the realm of possibility. Modern advancements in artificial intelligence and software design could potentially allow corrections to be calculated in the time it takes to capture or upload an image of the inadequately malpositioned radiograph.

## Conclusion

The present study aims to help surgeons understand the magnitude of LLD ME produced by increasing degrees of lateral PO and at varying degrees of femoral abduction and adduction. When the pelvis is level or at a PO of ≤5°, variations in femoral abduction or adduction of up to 15° produce errors of marginal clinical significance. As we approach a PO of 10° or 15°, even small changes in mediolateral limb position led to clinically significant LLD ME (> 1 cm). This study highlights the importance of proper patient positioning when obtaining radiographs for measurement of LLD and describes the relationship between true and functional LLD as affected by coronal pelvic and femoral orientation.

## Data Availability

All data generated or analyzed during this study are included in this published article.

## Abbreviations

AP: Anteroposterior
LLD: Leg length discrepancy
ME: Measurement error
PO: Pelvic obliquity
THA: Total hip arthroplasty
LCEA: Lateral center-edge angle
AI: Acetabular index
EI: Extrusion index
